# Immune thrombocytopenia with multi-organ dysfunction syndrome as a rare presentation of scrub typhus: a case report

**DOI:** 10.1186/s13104-017-2826-z

**Published:** 2017-10-06

**Authors:** Abraham M. Ittyachen, Saramma P. Abraham, Smitha Krishnamoorthy, Anuroopa Vijayan, Jayamohan Kokkat

**Affiliations:** 1 M.O.S.C Medical College & Hospital, Ernakulam District, Kolenchery, Kerala 682311 India; 2 Department of Anaesthesiology, M.O.S.C Medical College & Hospital, Ernakulam District, Kolenchery, Kerala 682311 India; 3 Department of Medicine, M.O.S.C Medical College & Hospital, Ernakulam District, Kolenchery, Kerala 682311 India

**Keywords:** Scrub typhus, Immune thrombocytopenia, Multi-organ dysfunction syndrome, Case report

## Abstract

**Background:**

Scrub typhus is an acute infectious illness caused by *Orientia tsutsugamushi*. It is endemic to a part of the world known as the “tsutsugamushi triangle”. Humans are accidental hosts in this zoonotic disease. About a third of patients admitted with scrub typhus have evidence of multi-organ dysfunction. Multi-organ dysfunction secondary to scrub typhus carries a high mortality rate.

**Case presentation:**

We report a 65-year old lady who was admitted in a Tertiary Care Center in the state of Kerala in India, with 7 day history of fever, myalgia and reduced urine output. Head to foot examination revealed the presence of an eschar on her chest. One week prior to the onset of her illness she had gone trekking through a hilly forest area. She was clinically suspected to have scrub typhus, which was later confirmed with laboratory tests. She developed multi-organ dysfunction syndrome secondary to this illness. Though there was an improvement in the multi-organ dysfunction, thrombocytopenia alone failed to improve. Bone marrow study was done which was suggestive of immune thrombocytopenia. Patient was given a course of steroids with which the thrombocytopenia improved.

**Conclusion:**

Failure of platelet count to normalize even after there has been a general improvement of other markers of multi-organ dysfunction in scrub typhus should prompt the clinician to consider other potential causes of thrombocytopenia. An unusual finding as this calls for further research to understand the molecular mechanisms behind such an event. Further, considering the close similarity in clinical presentation of several tropical illnesses, meticulous history taking and a detailed physical examination needs to be emphasized.

## Background

Scrub typhus is an acute infectious illness caused by *Orientia tsutsugamushi* [1]. It is transmitted to humans by an arthropod vector of the *Trombiculidae* family. Humans are accidental hosts in this zoonotic disease. Scrub typhus is endemic to a part of the world known as the “tsutsugamushi triangle”, which extends from northern Japan and far-eastern Russia in the north, to northern Australia in the south, and to India and Pakistan in the west [2]. The term ‘scrub’ is used because of the type of vegetation (terrain between woods and clearings) that harbors the vector. However the name is not entirely correct because certain endemic areas can be sandy, semi-arid and mountain deserts. Thus infection can be found in a wide variety of terrain [3].

Rickettsial diseases have been documented in India since the 1930s with initial reports being from the Kumaon region [4]. Scrub typhus gained notoriety only during the second world war when it caused several epidemics among the troops with resulting mortality and morbidity [5, 6]. Since then reports of this disease were rare. However recently there has been a resurgence of this disease with reports from almost all geographical regions of India [7–11].

The hallmark of scrub typhus is disseminated vasculitis [12] with resulting organ injury; organs that may be involved include the skin, liver, brain, kidney, meninges and the lung. The actual clinical manifestations range from non-specific febrile illness to severe organ dysfunction [13] in the form of disseminated intravascular coagulation (DIC), vascular leak, pulmonary edema, shock, hepatic dysfunction and meningoencephalitis. About a third of patients admitted with scrub typhus have evidence of multi-organ dysfunction [14].

Thrombocytopenia is a prominent feature of this disease and is usually part of the constellation of findings that make up the multi-organ dysfunction [15–17]. Several mechanisms have been elucidated to explain thrombocytopenia in multi-organ dysfunction [18, 19]. However a case of scrub typhus with multi-organ dysfunction and immune thrombocytopenia has not been described. Such a case is presented here.

## Case presentation

A 65-year old lady was admitted to a rural Tertiary Care Center in the state of Kerala in India, with 7 day history of fever, myalgia and reduced urine output. She did not have any significant medical history except for hysterectomy which she underwent 10 years ago for fibroid uterus. She was a widow and lived with her younger son. She neither smoked nor took alcohol.

**Fig. 1:**
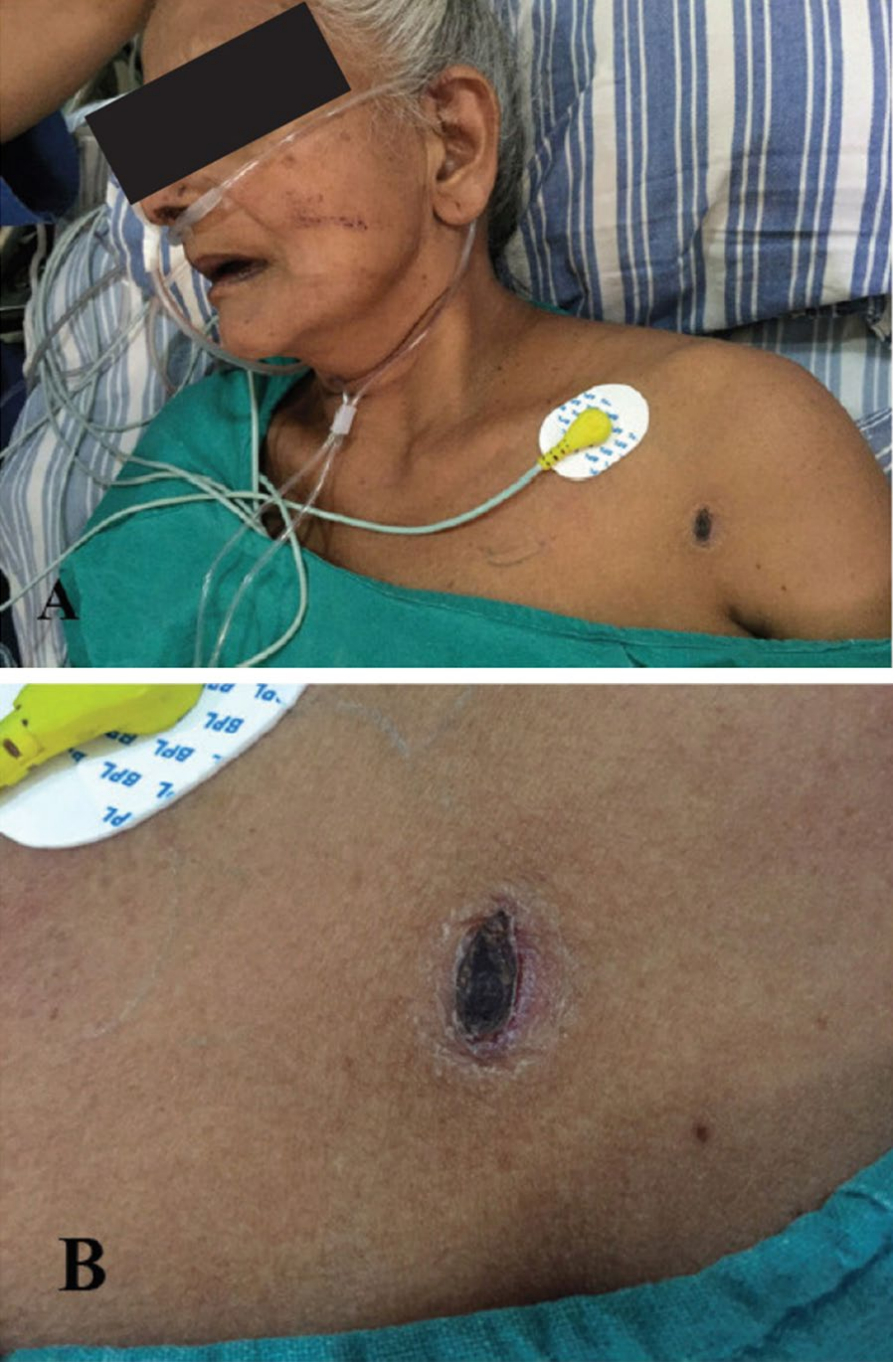
**A** Eschar, **B** magnified image of eschar

At the time of admission she was fully conscious and oriented. She was normotensive (blood pressure 120/70 mm of Hg) but tachycardic (pulse 120/min), tachypnoeic (respiratory rate 35/min) and febrile (38 °C). On auscultation of the chest there were bibasal crepitations. Head to foot examination revealed the presence of an eschar on the left side of the chest near the axilla (Fig. 1). At this juncture a review of her clinical history was done with the help of her son with whom the patient stayed. One week prior to the onset of her illness patient had gone for a pilgrimage which involved trekking through a hilly forest area. On the way she experienced an ‘insect bite’ on her shoulder but she chose to ignore it as it did not produce much symptoms.

Among her initial investigations haemoglobin was 10.9 g/dL, total white cell count 10.5 × 10^9^/L, (neutrophil count 8.1 × 10^9^/L, lymphocyte count 1.3 × 10^9^/L and monocyte count 6 × 10^9^/L) and platelet count 75 × 10^9^/L. Chest radiograph showed minimal infiltrates in the right lower zone. Liver enzymes were mildly elevated (serum aspartate aminotransferase 178; normal 14 to 36 U/L and serum alanine aminotransferase 272; normal 9 to 52 U/L) and so was the bilirubin level (total bilirubin 2.2; normal 0.2 to 1.2 mg/dL and direct bilirubin 1.6; normal 0 to 0.4 mg/dL). Urea, creatinine, amylase, lipase and electrolytes were normal initially. Based on a clinical suspicion of scrub typhus patient was initiated on doxycycline. As she came from an area that was endemic for leptospirosis and also had a risk for the same, penicillin was added subsequently.

In the next few days there was deterioration in her respiratory status with decrease in urine output. So she was put on non-invasive ventilation with bi-level positive airway pressure (BiPAP). However no improvement was noted in her respiratory status and on the fifth day of admission she had to be intubated and put on the ventilator. While on the ventilator she had repeated episodes of pulmonary oedema. Portable echocardiography showed regional wall motion abnormality with depressed left ventricular ejection fraction (< 35%). As her renal functions worsened (serum creatinine 4.3; normal 0.5 to 1.2 mg/dL) she was also initiated on dialysis. At this juncture Inj azithromycin was added to the treatment regimen.

In the meantime, confirming the clinical suspicion both Weil-felix test and Scrub typhus antibody was reported as positive in significant titres. IgM antibody to leptospira was however negative. Dengue, HIV and markers for Viral Hepatitis A and Viral Hepatitis B were also negative.

**Fig. 2:**
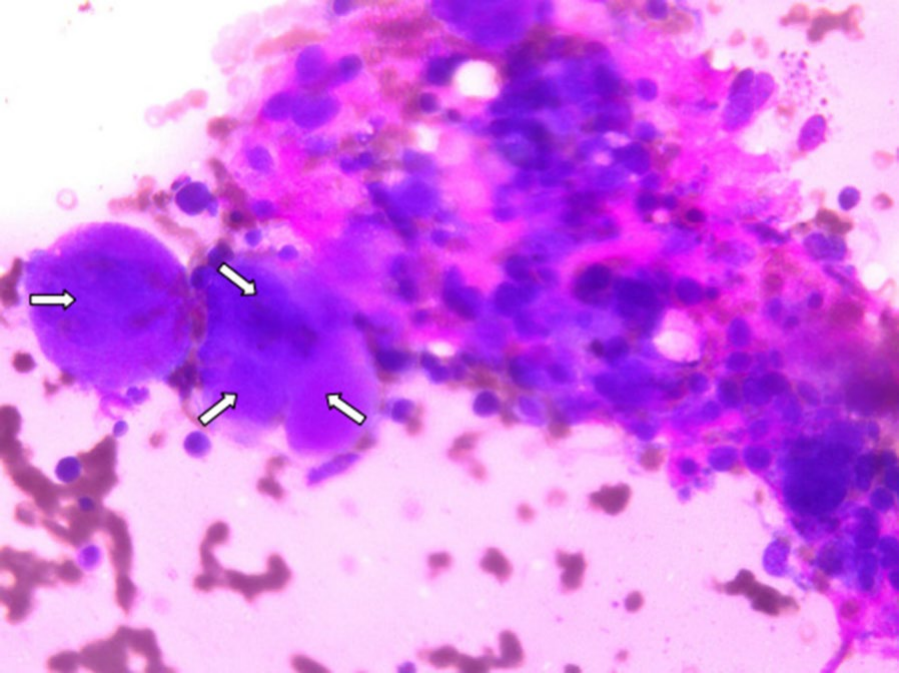
Cluster of megakaryocytes

After 8 days on the ventilator patient was extubated. Her renal functions improved and dialysis was discontinued. However she still had a persistent thrombocytopenia (platelet count 50 × 10^9^/L). Hence a bone marrow study was done which showed normal erythroid and myeloid series with megakaryocytic hyperplasia—reported by the pathologist as probable immune thrombocytopenia (Fig. 2). ANA was found to be positive but anti-ds DNA was reported as negative. Anti-platelet antibody was not checked as facilities were not available for the same. Patient was given a short course of steroids after which improvement in the platelet count was noted. She was discharged 20 days after admission. A repeat echocardiogram at discharge showed normal LV (left ventricular) systolic function. At follow up 1 week later she was back to her normal self and platelet count had also normalized.

## Discussion

Scrub typhus is one of the confirmed etiologies of acute febrile illness in tropical regions [20–23]. About a third of patients admitted with scrub typhus have evidence of multi-organ dysfunction [13]. Our patient had renal failure, myocarditis with severe LV dysfunction, hepatitis and thrombocytopenia. Multi-organ dysfunction secondary to scrub typhus carries a high mortality rate [24].

Several mechanisms have been elucidated to explain the thrombocytopenia in multi-organ dysfunction. Laboratory and clinical studies have confirmed that thrombocytopenia—associated multiple organ failure is a thrombotic microangiopathic syndrome defined by a variety of pathological changes that includes thrombotic thrombocytopenic purpura (TTP), secondary thrombotic microangiopathy (TMA), and disseminated intravascular coagulation (DIC) [18, 19]. However a case of scrub typhus with multiorgan dysfunction and immune thrombocytopenia has not been described. Neither has such a case been described from India.

There has been a resurgence of scrub typhus in India in recent years [25]. Similarity in clinical presentation to tropical illnesses that are already endemic to our region like leptospirosis, dengue, malaria, enteric fever and viral hepatitis makes early diagnosis a challenge [20]. A patient presenting with fever and myalgia in a tropical country (like this case) often tests the clinical acumen of a physician. Given the many differential diagnosis that may have similar clinical presentation there are limitations to clinical medicine. Also constraints in facilities for investigations in some countries compound the problem [26].

In light of the positive ANA result, there may have been some limitations in interpreting the clinical diagnosis in this patient. Quantitative testing (titre) was not available and the test was not repeated (sometimes repeat tests can be negative). Though unlikely, connective tissue disorders other than SLE cannot be totally excluded. Nevertheless soliciting a good history and undertaking a thorough physical examination (note the eschar [27] in this patient) helps the clinician to eliminate potential confounders and narrow down possibilities. This case further highlights the importance of intensive care in a case of tropical infection which can result in a favorable outcome.

## Conclusions

Given the close similarity in clinical presentation of several tropical illnesses, a meticulous history and a detailed physical examination needs to be emphasized.

Though thrombocytopenia is common in scrub typhus, failure of platelet count to normalize even when there has been a reversal of the other components of multi-organ dysfunction syndrome should prompt the clinician to consider other possibilities of thrombocytopenia notably immune thrombocytopenia. Considering the rarity of this finding there is also the need for further research to understand the molecular mechanisms behind such an event.

## Abbreviations

DIC: disseminated intravascular coagulation
BiPAP: bi-level positive airway pressure
ANA: anti-nuclear antibody
DNA: deoxyribonucleic acid
LV: left ventricular
TTP: thrombotic thrombocytopenic purpura
TMA: thrombotic microangiopathy
